# Different Pattern of Treatment Failure After Chemoradiation Between p16+ and p16− Patients Affected by Oropharyngeal Carcinoma

**DOI:** 10.1002/hed.70222

**Published:** 2026-03-05

**Authors:** Riccardo Gili, Paolo Bossi, Luca Lalli, Lisa Francesca Linda Licitra, Marta Maddalo, Almalina Bacigalupo, Liliana Belgioia, Jon Cacicedo, Nadia Facchinetti, Almudena Garcia Castano, Marc Oliva, Pierluigi Bonomo, Giuseppe Sanguineti, Pierfrancesco Franco, Athanassios Argiris, Amanda Psyrri, Ester Orlandi

**Affiliations:** ^1^ Medical Oncology Unit IRCCS Ospedale Policlinico San Martino Genoa Italy; ^2^ Medical Oncology, Department of Internal Medicine and Medical Specialties University of Genova Genoa Italy; ^3^ Department of Biomedical Sciences Humanitas University Milan Italy; ^4^ Unit of Translational Immunology IRCCS Foundation National Cancer Institute Milan Italy; ^5^ Fondazione IRCCS Istituto Nazionale dei Tumori and University of Milan Milan Italy; ^6^ Radiation Oncology Unit, Department of Medical and Surgical Specialties, Radiological Science and Public Health, ASST Spedali Civili of Brescia University of Brescia Brescia Italy; ^7^ Radiation Oncology Policlinico San Martino IRCCS Genoa Italy; ^8^ Department of Health Sciences University of Genova Genova Italy; ^9^ Department of Radiotherapy Cruces University Hospital. Osakidetza, Basque Health Service Barakaldo Spain; ^10^ Radiation Oncology Clinical Department National Center for Oncological Hadrontherapy (CNAO) Pavia Italy; ^11^ Medical Oncology Department Hospital Universitario Marqués de Valdecilla Santander Spain; ^12^ Institut Català d'Oncologia L'Hospitalet Institut d'Investigació Biomèdica de Bellvitge Barcelona Italy; ^13^ Radiation Oncology Azienda Ospedaliero Universitaria Careggi Florence Italy; ^14^ Department of Radiation Oncology IRCCS Regina Elena National Cancer Institute Rome Italy; ^15^ Department of Radiation Oncology 'Maggiore della Carità' University Hospital Novara Italy; ^16^ Department of Translational Medicine (DIMET) University of Eastern Piedmont Novara Italy; ^17^ Sidney Kimmel Comprehensive Cancer Center Thomas Jefferson University Philadelphia Pennsylvania USA; ^18^ Section of Medical Oncology, Department of Internal Medicine, Faculty of Medicine, National and Kapodistrian University of Athens Attikon University Hospital Athens Greece; ^19^ Department of Clinical, Surgical, Diagnostic, and Pediatric Sciences University of Pavia Pavia Italy; ^20^ Radiation Oncology Unit Fondazione IRCCS Istituto Nazionale dei Tumori Milan Italy

**Keywords:** chemoradiotherapy outcomes, HPV/p16 status, oropharyngeal squamous cell carcinoma, recurrence patterns, smoking history

## Abstract

**Background:**

Oropharyngeal cancer (OPC) exhibits distinct clinical behaviors according to HPV/p16 status and smoking exposure. While HPV‐positive OPC generally shows superior survival, differences in recurrence patterns remain unclear.

**Methods:**

A retrospective multicenter analysis of 674 AJCC VII edition Stage III–IVa/b OPC patients treated with definitive IMRT ± systemic therapy was conducted across 14 Southern European centers. Recurrence patterns, disease‐free survival (DFS), overall survival (OS), and survival after recurrence (rOS) were compared between p16 groups and across smoking categories.

**Results:**

HPV‐positive tumors showed a higher incidence of distant recurrence, particularly among heavy smokers, while p16‐negative OPC displayed predominantly locoregional failure. p16‐positive patients had significantly better DFS, OS, and rOS.

**Conclusion:**

Recurrence patterns in OPC differ by HPV status and smoking history. HPV‐positive heavy smokers represent a distinct high‐risk subgroup characterized by increased distant metastasis and inferior survival, warranting refined stratification beyond p16 status alone.

## 1 Introduction

Head and neck squamous cell carcinoma (HNSCC) is a heterogenous group of tumors that arise from the oral cavity, oropharynx, nasopharynx, hypopharynx, or larynx. It's the sixth most commonly diagnosed cancer worldwide [1], with approximately 800 000 new cases and 400 000 deaths annually [2]. The highest incidence occurs among males aged 50–70 years, although the geographic distribution of specific subsites varies significantly [3].

However, in recent years, there has been a notable increase of oropharyngeal cancer (OPC), including base of tongue, palatine tonsil, soft palate, or oropharyngeal wall, due to the increasing rates of infection with human papillomavirus (HPV) [4].

An estimated 20%–40% of OPC is believed to be caused by HPV infection, and 80% of them are due to HPV16 [5].

To verify the association between HPV and OPC, detection methods combining p16 immunohistochemistry and HPV‐DNA testing are recommended [6]. Almost the entirety of p16− patients are strongly associated with tobacco (> 10 p/y and alcohol use), while p16+ OPCs have risk factors related to sexual behavior [7].

P16+ patients, despite having more extensive nodal disease at presentations, show substantially better survival rates compared to p16− patients, along with a lower overall relapse rate and a longer disease‐free survival (DFS) [8]. If compared to HPV‐, the improved survival of HPV+ OPSCC patients is well described, but the difference in the pattern of recurrence between p16+ and p16− patients has been less clearly investigated. If compared with p16− patients, p16+ OPCs have a reduced rate of local/locoregional (LR), but the distant relapse (DR) incidence is still unclear [9, 10, 11, 12, 13, 14, 15, 16]. In addition, several studies have reported that p16+ OPCs are more likely to metastasize to multiple and atypical distant organs [9].

Moreover, regarding survival after disease recurrence, data suggest that p16+ patients have a better overall survival (OS) [9].

This manuscript is the first one to describe the different patterns of failure in a large cohort of OPCs from Southern Europe, where large prospective studies—such as the de‐escalation ones—have not been performed, differently from North America or Northern Europe, and aims to analyze and compare the pattern of failure after primary chemoradiation by p16 status in a large cohort of consecutively treated patients at 14 Southern European Centers.

## 2 Materials and Methods

A large retrospective review from consecutive series of locally‐advanced AJCC VII ed. Stage III–IVa/b OPC from 14 Southern European Oncology Units (Italy, Greece, Spain) treated with definitive radiotherapy (RT) with or without systemic treatments was performed.

All patients underwent a complete medical history and imaging (computed tomography‐scan or magnetic resonance for head and neck, computed tomography‐scan or positron emission tomography scan for distant sites) before treatment starting. HPV status was determined by p16 immunohistochemical analysis with strong and diffuse staining in > 70% of tumor cells considered positive and a confirmatory test—HPV DNA‐ish for example—was allowed. Significant smoking history was defined as a total of more than 10 pack‐years. Patients with unknown p16 status were excluded from the analysis.

All patients undergoing primary RT were treated with intensity modulated radiation therapy (IMRT) with fixed‐gantry or rotational techniques (tomotherapy or volumetric modulated arc therapy [VMAT]), and generally received 70 Gy/35 fractions (5 daily fractions/week) to gross disease plus appropriate margins. Patients receiving chemoradiotherapy (CRT) were treated with concurrent high‐dose cisplatin (100 mg/m^2^ on Days 1, 22, and 43) or weekly cisplatin 40 mg/m^2^. Other possible treatments were concomitant carboplatin (tri‐weekly or weekly) and cetuximab.

Local recurrence was defined as the presence of tumor at the primary site at least 6–8 weeks after primary treatment completion; regional recurrence as the presence of tumor at the lymph nodes of the neck, and distant metastases as the presence of tumor in an organ outside the head and neck district. Patients with local and/or regional recurrence were considered as patients with locoregional relapse. DFS was defined as the time from the beginning of treatment to the diagnosis of recurrence. Overall survival (OS) was calculated from the date of the start of treatment to the date of death or last follow‐up, while disease from recurrence (rOS) was calculated from the date of recurrence/metastases to the date of death or last follow‐up.

A second primary tumor (SPT) was defined as a malignancy distinct from the index tumor, occurring in a different anatomical site or presenting histopathological features incompatible with metastatic disease from the primary tumor. The SPT diagnosis required histological confirmation whenever feasible. In cases in which tissue biopsy was not technically feasible or was contraindicated due to patient comorbidities or procedural risk, a diagnosis based solely on radiological findings was accepted, provided that imaging features were deemed unequivocally diagnostic and sufficient to discriminate a SPT from disease progression or metastasis of the index tumor.

## 3 Statistical Analyses

Kaplan–Meier curves and Log‐rank tests were used to analyze DFS, OS, and rOS. Kaplan–Meier analyses and Cox proportional hazards regression models were also stratified by clinical variables of interest to further investigate the impact of these factors on survival outcomes.

Prognostic factors were investigated through univariate analyses using the Cox proportional hazards regression models. The proportional hazards (pH) assumption was checked to ensure model validity.

To evaluate statistical significance of the associations between categorical variables, the chi‐square test was used: the conventional two‐sided 5% level was chosen as the threshold.

All statistical analyses were performed with R software (version 4.3.3, R Foundation for Statistical Computing, Vienna, Austria).

## 4 Results

### 4.1 Different Patterns of Recurrence

From a total of 805 patients identified with OPC and treated with primary RT with or without concurrent chemotherapy (CT), 131 patients were excluded due to unknown p16− status as well as smoking history.

Of the final 674 patients, 249 were p16− (36.9%) and 425 were p16+ (63.1%).

Considering the 425 p16+ patients, 214 (50.4%) were heavy smokers (> 10 packs/year), 65 (15.3%) light smokers (< 10 packs/year), and 146 (34.3%) never smokers.

For the p16− group, the heavy smokers were 206 (82.7%), while the light and never smokers were respectively 14 (5.6%) and 29 (11.7%).

One hundred and nine out of 249 patients (43.8%) from the p16− group experienced recurrence/metastasis versus the 78 out of 425 pts. (18.4%) from the p16+ group.

Different patterns of recurrence are reported in Table 1.

**TABLE 1:** Patients' characteristics and different patterns of recurrence.

	p16−	p16+ total	p16+ NS	p16+ < 10 PY	p16+ (NS + < 10 PY)	p16+ ≥ 10 PY
n° pts	249	425	146	65	211	214
Recurrence	109	78	21	11	32	46
Exclusive DR	31/109–28.4%	30/78–38.5%	6/21–28.6%	4/11–36.4%	10/32–31.3%	20/46–43.5%
Exclusive DR and both DR + LR	39/109–35.8%	40/78–51.3%	8/21–38.1%	5/11–45.5%	13/32–40.6%	27/46–58.7%
Exclusive LR	70/109–64.2%	38/78–48.7%	13/21–61.9%	6/11–54.5%	19/32–59.4%	19/46–41.3%
Exclusive LR and both DR + LR	78/109–71.6%	48/78–61.5%	15/21–71.4%	7/11–63.6%	22/32–68.7%	26/46 56.5%

### 4.2 Distant Recurrence

In the p16+ cohort the incidence of isolated DR was 38.5% (30 out of 78 patients), while 40 patients (51.3%) developed DR alone or combined with LR (10 patients developed both DR and LR). For the p16− cohort, patients who developed exclusively DR were 28.4% (31 out of 109) and 39 patients (35.8%) experienced DR alone or combined with LR (eight patients developed both DR and LR).

### 4.3 Local/Locoregional Recurrence

In the p16− cohort the incidence of exclusive LR was 64.2% (70 out of 109 patients), while 78 patients (71.6%) developed LR alone or combined with DR. For the p16+ cohort, patients who developed exclusively LR were 48.7% (38 out of 78) and 48 patients (61.5%) experienced LR alone or combined with DR.

### 4.4 P16+ and Smoking Status

Considering p16+ patients, a subgroup analysis was performed according to smoking history (> 10 p/y, < 10 p/y, and never smokers). For p16+ never smokers and light smokers, patients who experienced a recurrence were 21 (out of 146 patients, 14.4%) and 11 (out of 65 patients, 16.9%) respectively, while 46 (out of 214 patients, 21.5%) recurrences were observed in the p16+ heavy smokers group. The incidence of exclusively DR and DR alone or combined with LR was higher in the heavy smokers group (43.5% and 58.7%) than what was observed in the light (36.4% and 45.5%) and never smokers (28.6% and 38.1%) groups.

On the contrary, the heavy smokers group experienced lower rates of exclusive LR and total LR (also combined with DR) incidence (41.3% and 56.5%) when compared with light (54.5% and 63.6%) and never smokers (61.9% and 71.4%).

Significant associations were observed in the comparisons of p16+ total versus p16− and p16+ > 10 pack‐years vs. p16−, particularly in the DR ± LR and exclusive LR groups (*p* < 0.05). No significant differences were found in the comparison between p16+ > 10 pack‐years and p16+ (non‐smoker + < 10 pack‐years). Chi‐square test results are reported in Table 2.

**TABLE 2:** Chi‐square tests between different subgroups according to the p16 status and smoking history.

Comparison	Exclusive DR	DR ± LR	Exclusive LR	LR ± DR
	*p*			
p16+ total vs. p16−	0.149	0.034	0.032	0.143
p16+ > 10 p/y vs. p16−	0.068	0.008	0.007	0.551
p16+ > 10 p/y vs. other p16+	0.275	0.116	0.126	0.221

### 4.5 Disease Free Survival and Overall Survival

#### 4.5.1 DFS

**FIGURE 1:**
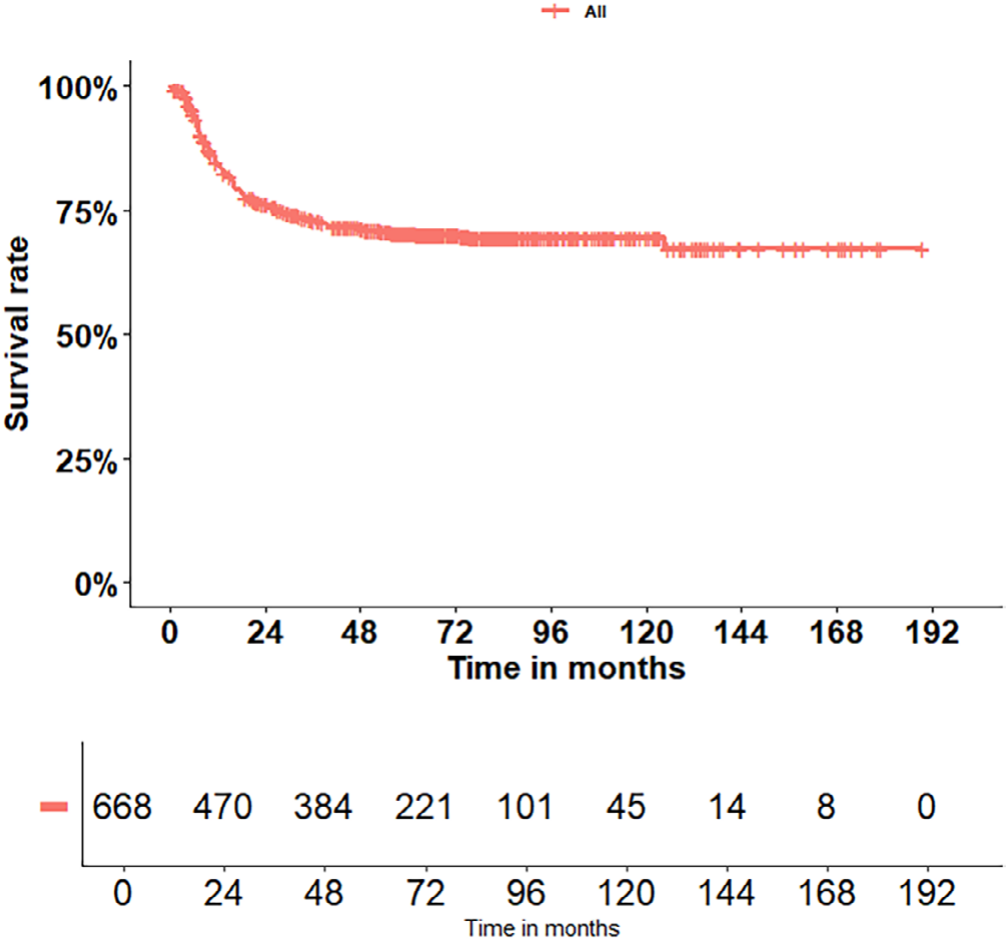
Overall DFI. Median DFI was not reached. [Color figure can be viewed at wileyonlinelibrary.com]

From the 668 patients with complete data for DFS evaluation, 247 were p16− and were 421 p16+. The overall 5‐year DFS was 70.41% (CI: 66.91–74.1), with a median DFS not yet reached (Figure 1).

**FIGURE 2:**
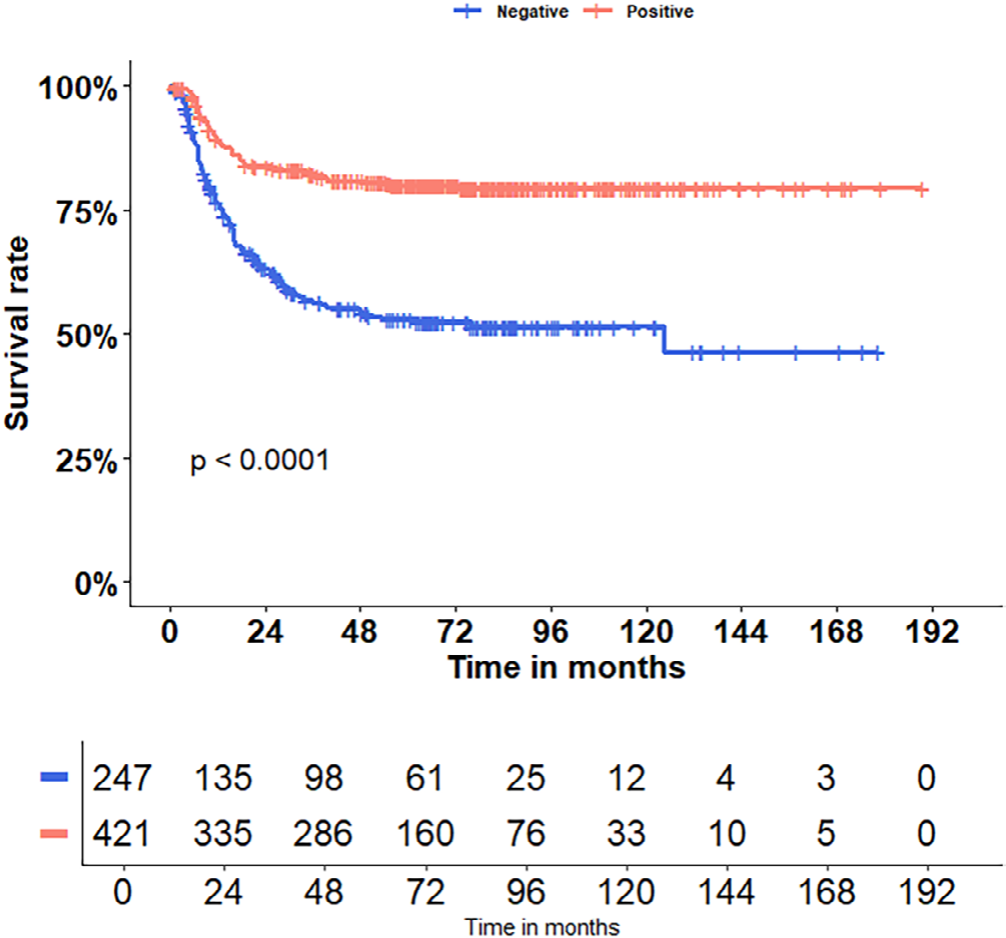
DFI according to the p16− status. [Color figure can be viewed at wileyonlinelibrary.com]

When compared by p16 status, 5‐year DFS was 80.04% for p16+ patients (CI: 76.21–84.07), significantly better than 53.02% observed for the p16− group (CI: 46.78–60.09) (HR 0.3469, 95% CI 0.2621–0.4665, p 0.0001) (Figure 2). While for the p16− cohort the median DFS was 124.35 months, for the p16+ group it was not reached.

For p16+ patients, a subgroup analysis was conducted based on smoking history. All three subgroups showed a better DFS than that observed in p16− patients, without achieving a median DFS.

**FIGURE 3:**
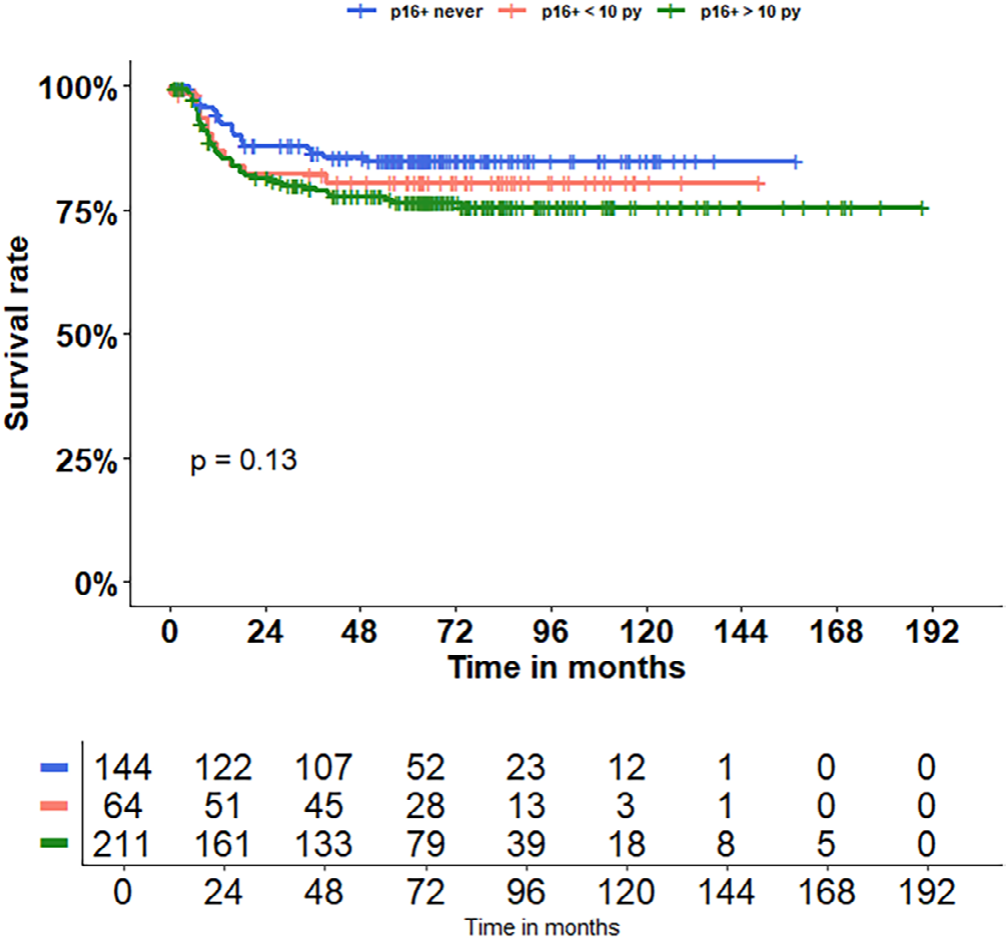
DFI for the p16‐subgroups according to the smoking status. [Color figure can be viewed at wileyonlinelibrary.com]

The never‐smokers 5‐year DFS was 85.01% (CI: 79.29–91.15), 80.62% (CI: 71.35–91.1) (HR 1.3543, 95% CI 0.6663–2.7528, p 0.4020), and 76.58% (CI: 70.86–82.77) (HR 1.6881, 95% CI 1.0091–2.8240, p 0.0461) for light smokers and heavy smokers respectively (Figure 3).

#### 4.5.2 OS

From the 671 patients with complete data for OS evaluation, there were 246 p16− and 425 p16+ patients.

**FIGURE 4:**
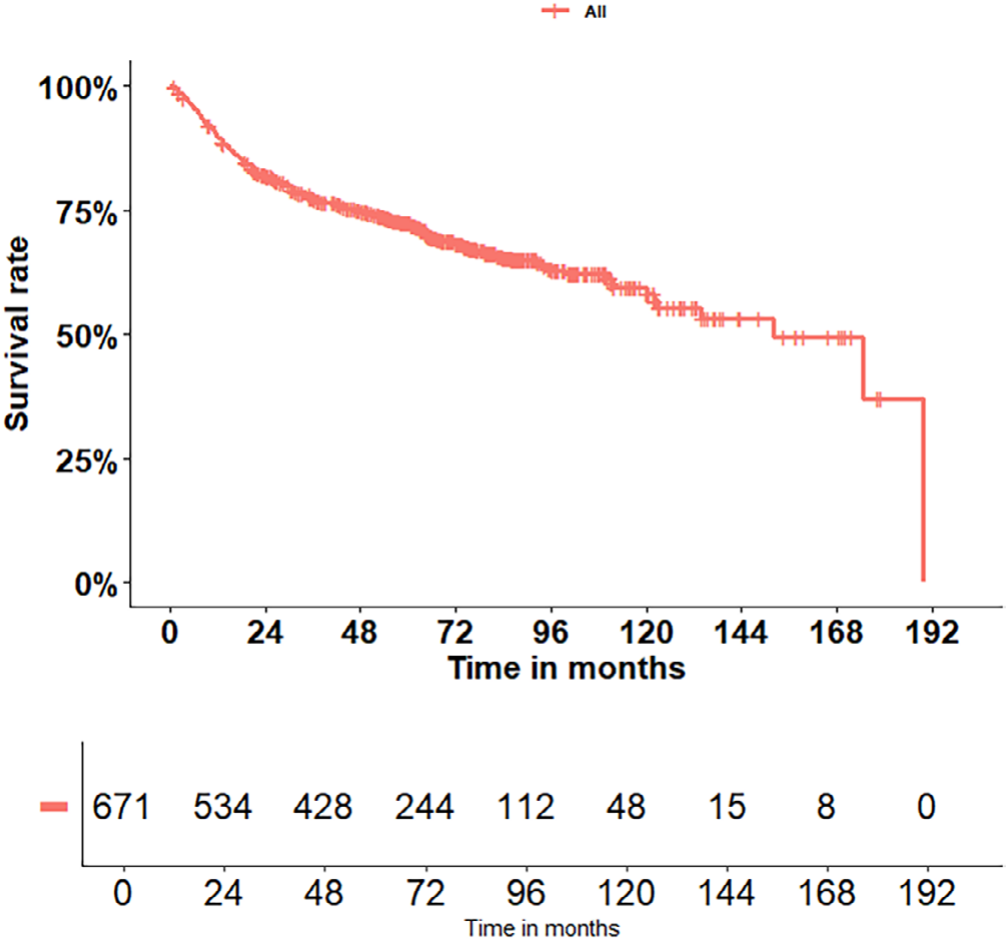
Overall survival. [Color figure can be viewed at wileyonlinelibrary.com]

The overall 5‐year OS was 72.62% (CI: 69.23–76.17), with a median OS of 152.11 months (Figure 4).

As observed for DFS, p16+ patients also had a better outcome.

**FIGURE 5:**
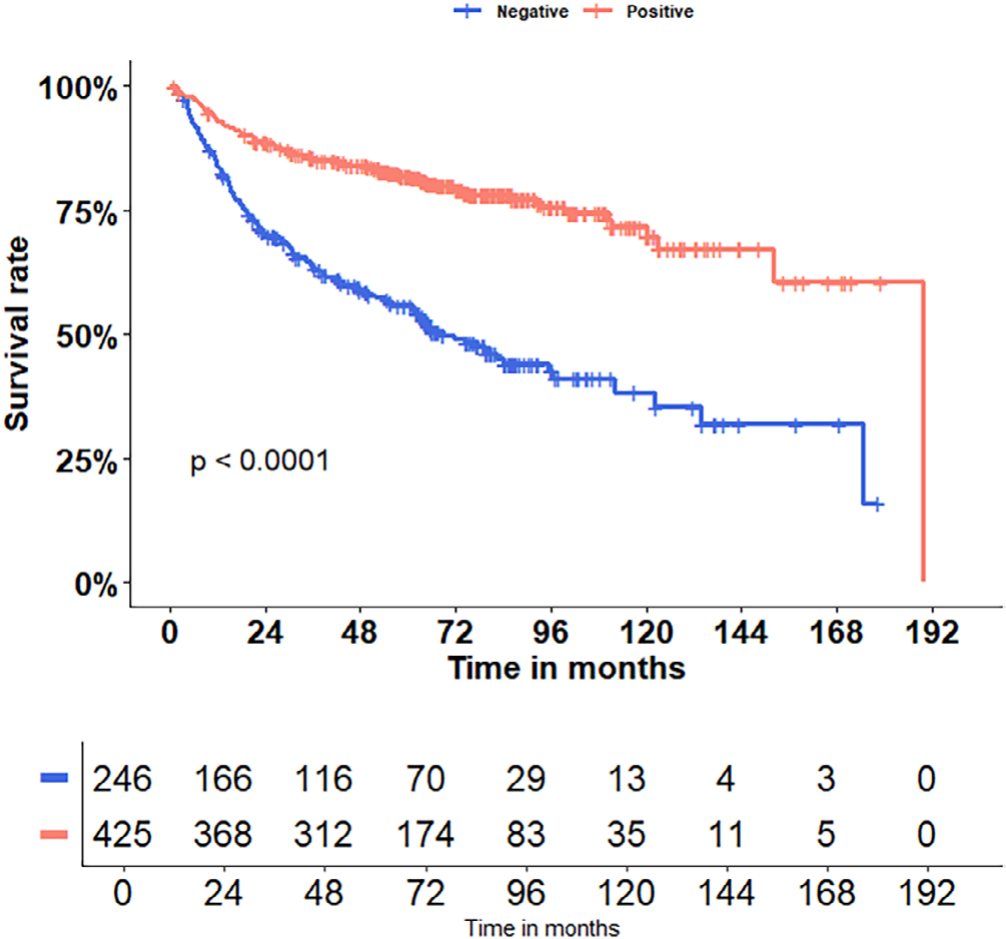
Overall survival according to the p16− status. [Color figure can be viewed at wileyonlinelibrary.com]

The p16+ 5‐year OS was 82.09% (CI: 78.43–85.92), being greater than 55.85% (CI: 49.74–62.71) observed for the p16− cohort (HR 0.3383, 95% CI 0.2583–0.4430, p 0.0001), with a median OS of 189.73 months and 68.73, respectively (Figure 5).

**FIGURE 6:**
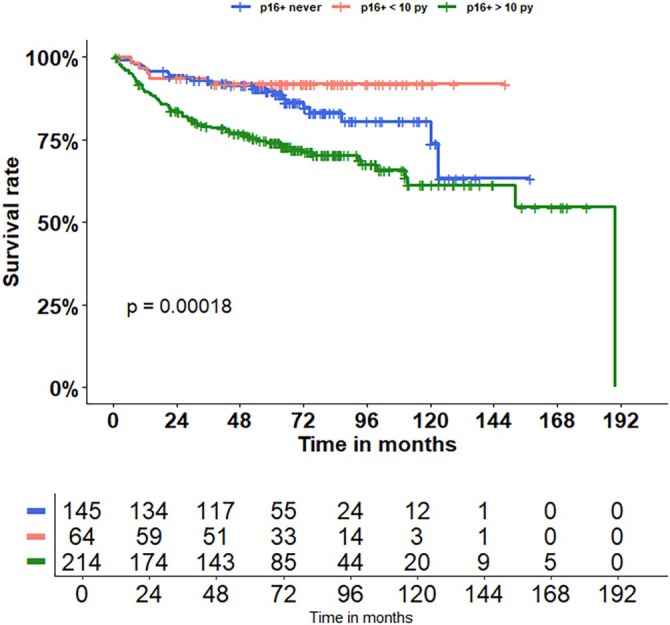
Overall survival for the p16‐subgroups according to the smoking status. [Color figure can be viewed at wileyonlinelibrary.com]

In the subgroups analysis, the 5‐year OS was 89.77% (CI: 84.8–95.04) for the never smokers, 92.01% (CI: 85.53–98.98) for the light smokers (HR 0.5062, 95% CI 0.1916–1.3370, p 0.1695), and 74.17% (CI: 68.38–80.45) for the heavy smokers (HR 2.0691, 95% CI 1.2731–3.3626, p 0.0033) (Figure 6). The median OS was achieved only for the heavy smokers cohort (189.73 months).

**FIGURE 7:**
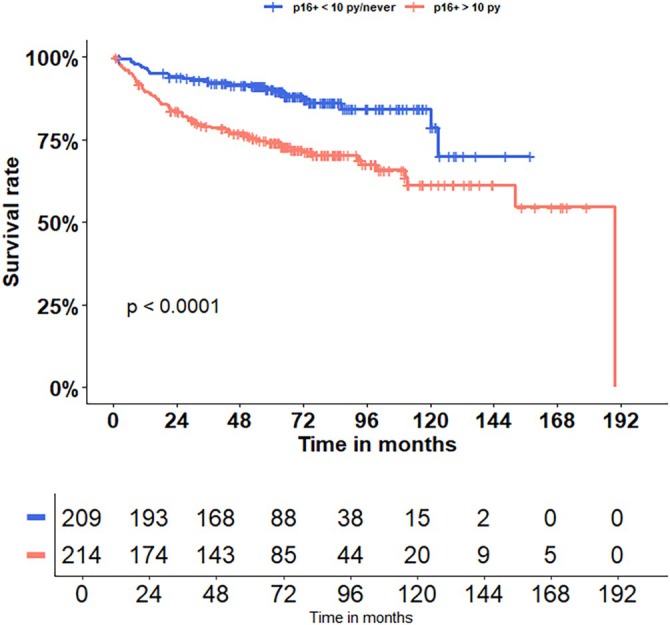
Overall survival for p16+ heavy smokers versus light + never smokers. [Color figure can be viewed at wileyonlinelibrary.com]

To accurately differentiate p16+ heavy smokers, p16+ light and never smokers have been grouped together with a 5‐year OS of 90.48% (CI: 86.48–94.67), superior to that observed in the heavy smokers group (HR 2.4426, 95% CI 1.5557–3.8352, p 0.0001), whereas median OS was not reached (Figure 7).

### 4.6 OS After Recurrence

For the 185 patients who experienced LR or DR recurrence and for whom data were available, the time from diagnosis to death or last follow‐up, defined as rOS, was evaluated.

**FIGURE 8:**
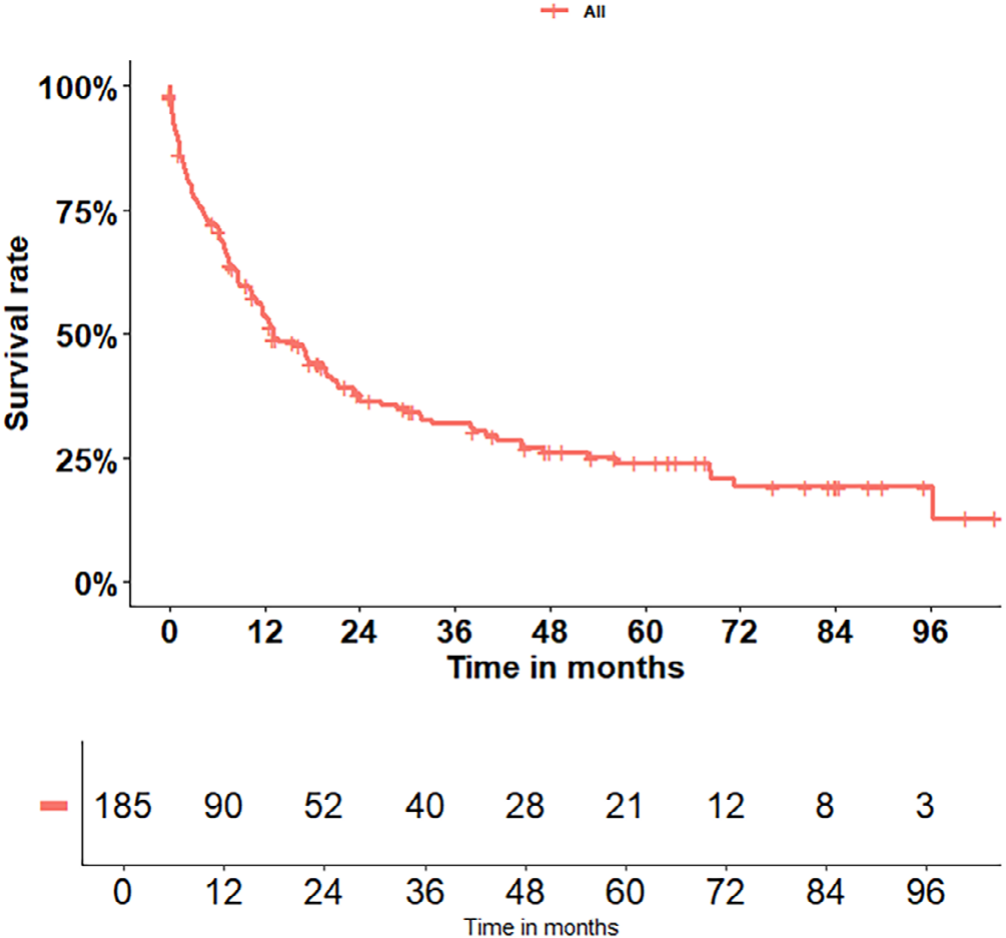
Survival after recurrence. [Color figure can be viewed at wileyonlinelibrary.com]

The overall 3‐year rOS was 31.93% (CI: 25.4–40.14), and the median rOS was 13.11 months (Figure 8).

**FIGURE 9:**
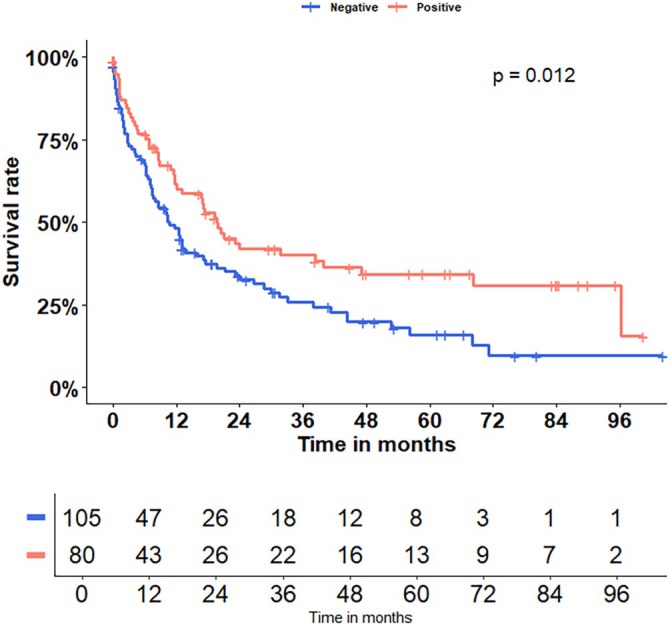
rOS according to the p16− status. [Color figure can be viewed at wileyonlinelibrary.com]

For the 105 p16− patients, the median rOS was 10.64 months and the 3‐year rOS was 25.77% (CI: 18.05–36.78), being significantly worse than observed for p16+ patients, where the median rOS and 3‐year rOS were 19.68 months and 40.01% (CI: 29.9–53.53), respectively (HR 0.6329, 95% CI 0.4416–0.9071, p 0.0127) (Figure 9).

**FIGURE 10:**
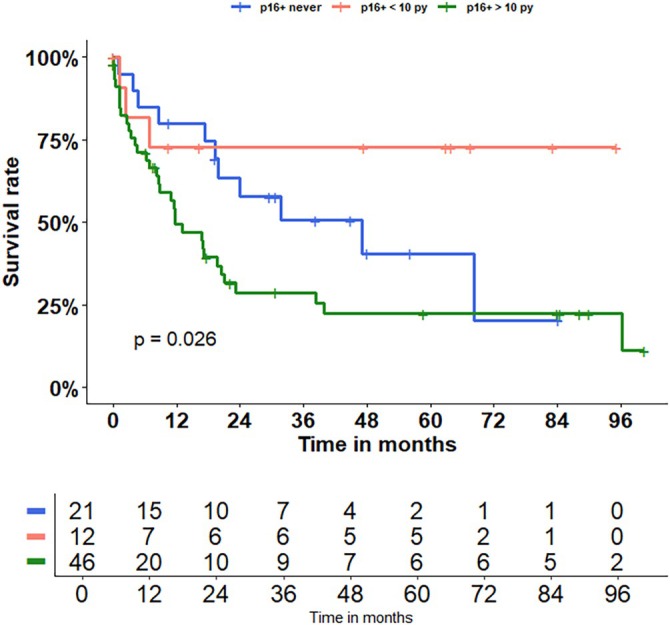
Overall survival for the p16‐subgroups according the smoking status. [Color figure can be viewed at wileyonlinelibrary.com]

Similarly to the OS analyses for the three subgroups of p16+ cohort, light smokers showed better median rOS (not reached) and 3‐year rOS 72.73%, (CI: 50.64–100) (HR 0.5018, 95% CI 0.1396–1.8035, p 0.2908), when compared with heavy smokers (median rOS 11.73 months, 3‐year rOS 28.68%, CI: 17.58–46.81) (HR 1.8531, 95% CI 0.9303–3.6915, p 0.0794) and never smokers (median rOS 47.11 months, 3‐year OS 50.56%, CI: 31.63–80.81) (Figure 10).

**FIGURE 11:**
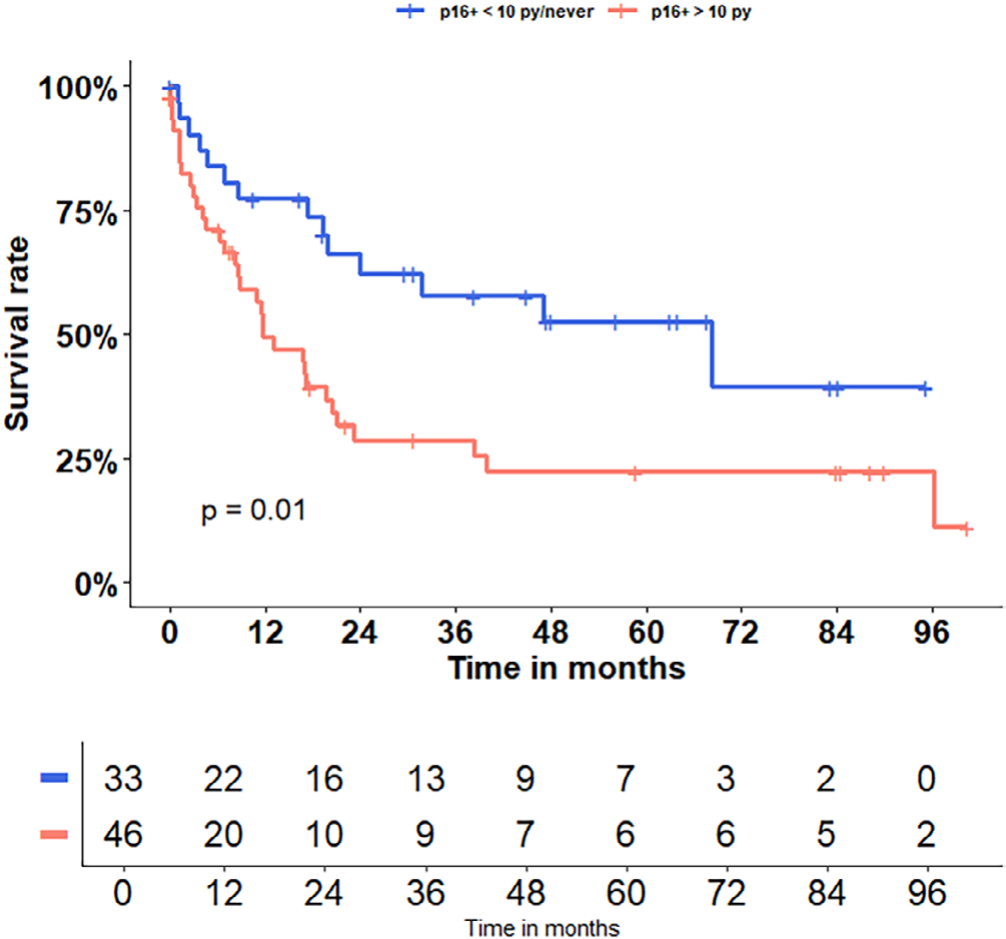
rOS for p16+ heavy smokers versus light + never smokers. [Color figure can be viewed at wileyonlinelibrary.com]

When never smokers and light smokers subgroups were combined, the median rOS was 68.34 months, with a 3‐year rOS of 57.82% (CI: 41.93–79.72) (Figure 11).

### 4.7 Second Primary Tumor

During the entire follow‐up period, 78 out of 684 patients (11.4%) developed a SPT. The most frequently involved sites were the head and neck region (*n* = 20) and the lung (*n* = 15), followed by the esophagus (*n* = 7), the gastrointestinal tract including stomach and colon (*n* = 12), and the genitourinary system (*n* = 7). The remaining 17 SPTs involved other organs, none of which accounted for more than five cases. These included pancreatic, hepatic, cutaneous, ocular, and breast malignancies, as well as sarcomas and cholangiocarcinomas.

## 5 Discussion

We evaluated a large cohort of patients from European institutions with locally advanced OPC treated with CRT to assess their possible DPR, and we observed significant differences between p16+ and p16− patients. We did not consider just the first recurrence but the entire follow‐up has been assessed to obtain a broader and more detailed understanding of the different subgroups' behavior. In the p16− subgroup, the overall probability of recurrent disease was higher, in accordance with a more aggressive behavior and worse prognosis compared to the p16+ counterpart, as already known. However, when evaluating recurrence patterns, p16‐positive patients exhibited a relatively higher rate of DR, with a difference exceeding 15% when accounting for both isolated DR (*p* = 0.149) and the combination of DR ± LR (*p* = 0.034). In contrast, p16− patients experienced a higher LR rate (*p* = 0.032) and LR ± DR (*p* = 0.149). While this increased LR rate among p16‐negative patients is consistent with previously reported findings in the literature [9, 10, 11, 12, 13, 14, 15, 16], the main available data had never shown a higher relative incidence of DR for p16+ patients [9, 10, 11, 12, 13, 14, 15, 16].

The increased incidence of LR in p16− patients may have several justifications:

1. In this study, p16− patients had more advanced primary stage disease than the p16+ group;
2. As already known in the literature, HPV/p16‐negative patients are more radioresistant, due to a different DNA repair capacity [17, 18];
3. The vast majority of p16‐negative patients were smokers. A more extensive smoking history, particularly when accompanied by a shorter interval between smoking cessation and the initiation of radiotherapy, is associated with increased cellular hypoxia. This hypoxic environment is known to correlate directly with enhanced radioresistance, thereby potentially compromising therapeutic outcomes [18].

However, this factor alone is insufficient to comprehensively address the questions surrounding DPR.

A sub‐analysis of p16‐positive patients stratified by smoking history revealed that the heavier smoker subgroup deviates significantly from the p16‐negative group. When evaluating recurrence patterns and compared with p16‐negative patients, the heavy smoker subgroup exhibited a higher rate of isolated DR (*p* = 0.068) and DR ± LR (*p* = 0.008), showing the greatest incidence of DR among all subgroups. In contrast, light and never smoker subgroups exhibit a recurrence profile more closely resembling that of p16‐negative patients. Data available in the literature [9, 13] have shown no difference regarding the incidence of DR between p16+ and p16− patients, but a higher number of sites and a greater number of lesions were detected in the p16+ group, although the authors could not give a justification for it.

Several reasons could justify the DPR observed in our study, including the interaction between tumor characteristics and its microenvironment (TME) and the voluptuary habits' role.

Referring to the TME, HPV‐associated OPCs is a distinct clinical entity when compared with HPV‐negative OPCs, particularly in HPV16+ OPCs patients with a type 1‐oriented intratumoral T cell response [19]. Strong infiltration by CD8+ and CD4+ T cells in pretreatment OPCs is associated with lower T stage, improved disease specific survival (DSS) and prolonged OS. A higher frequency of CD161+ CD4+ T cells is related with prolonged survival, and also the macrophages recruitment is higher in HPV+/p16+ patients [20].

HPV16+ tumors can evoke immune responses, expressing the virally derived oncoproteins E6 and E7. Cellular and humoral immunity against these antigens can be detected both in the peripheral blood and TME of HPV16+ OPC patients, with a direct link between the presence of an intratumoral HPV‐specific T‐cell response and the good prognosis of HPV16‐driven OPSCC [20].

Unfortunately, the available data on TME, while confirming its important prognostic and predictive role, do not provide data between the TME characteristics and the DPR, especially regarding the propensity to develop distant metastases and the tropism for specific anatomical districts.

In addition, despite we have observed significant differences in the pattern of recurrence between the various smoking‐based subgroups of p16+ OPCs, just a few data regarding possible different patterns of TME based on the smoking history are available.

Smoking is known to play a prognostic role, acting on cellular proliferation, invasion, metastasis, migration, angiogenesis [21], and in HNSCCs in particular has an immunosuppressive effect through inhibition of tumor infiltration of cytotoxic T‐cells, likely as a result of suppression of IFN response pathways [22, 23].

COX‐2 expression and activity is often induced by cigarette smoke exposure, which also increases the release of PGE2 and TxA2, leading to an imbalance in PGI2 and TxA2 production in favor of the latter. It has pro‐inflammatory effects in a PGE2‐dependent way, contributing to carcinogenesis and tumor progression [24].

Inflammation has already been shown in HNSCCs to be one of the factors influencing progression and metastatising by VEGF, PI3K/AKT/mTOR, STAT, Cyclooxygenase (COX)‐2 Pathway [25]. In particular, COX‐2‐dependent E‐cadherin expression is regulated by the inflammatory mediator IL‐1β that modulates Snail, and Snail overexpressing cells significantly increased metastatic (and primary) tumor burdens in HNSCC [26].

Zevallos et al. showed alterations in the smoking HPV‐positive OPC TME, characterized primarily by reduced cytolytic activity, decreased immune infiltration, and IFN‐gamma pathway signaling [27].

However, evidence regarding differences in the mutational profile between HPV‐positive OPC smokers and non‐smokers remains poor and discordant. Lassen et al. reported an increased frequency of mutations in CDKN2A, KRAS, NOTCH1, and TP53, along with a decrease in HLA‐A mutations among smokers [28]. In contrast, Mirghani et al. did not observe any significant differences [29].

Evidence of how smoking promotes DR can also be found in other malignancies.

For example, nicotine promotes breast cancer metastasis by stimulating N2 neutrophils and generating pre‐metastatic niche in lung [30], smoking induced acute and active inflammation contribute to the pathogenesis and development of pulmonary metastasis in colorectal cancer, and current smoking at diagnosis is a risk factor for developing distant metastasis in prostate cancer patients [31].

As seen above, in order to better predict the different patterns of recurrence, it will be necessary to better understand how TME varies depending on the HPV/p16 status and smoking history, and consequently what mechanisms favor distant metastatization.

With regard to the survival data obtained, we found no substantial differences with the literature data available now. While overall p16+ patients have a better prognosis in terms of DFS and OS than p16− patients, evaluating the subgroups based on smoking history for the HPV‐related population, it is confirmed that there is a worse prognosis. as smoking history increases.

A more interesting finding emerged from the rOS, defined as survival from the time of first recurrence. While it was already known in the literature that despite being aggressive diseases, at recurrence (irrespective of whether LR or DR) the prognosis was better in the HPV‐related cohort, there was no clear data concerning the division of p16+ patients according to their smoking history.

Our study showed that all p16+ patients have a better rOS than p16− patients, but, as seen for OS and DFS, heavy smokers have a worse prognosis than light and never smokers, indicating that in addition to p16‐status, smoking history also has a major impact on rOS, and categorizing patients only by p16‐status is no longer enough.

We acknowledge several limitations. We cannot exclude the potential for bias in our model because of the exclusion of a proportion of enrolled patients as a result of missing data for key variables. Secondarily, we included AJCC 7th edition T stage and N stage categories for OPCs, adding a possible staging bias.

In addition, this was a retrospective study, with no standardized follow‐up strategy in terms of type and frequency of imaging assessments, and it is possible that smokers underwent a higher number of imaging examinations aimed at detecting smoking‐related second primary tumors.

In our analysis, data on second primary tumors were collected and diagnoses were established based on histological confirmation whenever feasible, or on imaging findings when biopsy was not possible and radiological features were deemed unequivocally diagnostic. The same diagnostic approach was applied to distant lesions classified as metastases, in order to reliably distinguish metastatic disease from second primary tumors.

Finally, data regarding progression‐free interval and detailed patterns of distant recurrence—such as number of lesions and number or type of involved sites—were not available. Although this information would have been helpful to further characterize disease behavior, aggressiveness, and patterns of tumor spread among the different groups (p16‐positive vs. p16‐negative patients and among p16‐positive subgroups), the high level of diagnostic confidence used to differentiate second primary tumors from metastases makes a substantial misclassification unlikely. Therefore, it is difficult to assume that an incorrect classification of second primary tumors as metastatic disease may have significantly influenced our results.

## 6 Conclusions

OPCs have shown different patterns of recurrence according to the HPV status and the smoking history. HPV‐positive OPC experienced more DR when compared with HPV‐negative OPC, which, in contrast, had a higher rate of LR.

Considering the different HPV‐positive subgroups depending on the smoking history, heavy smokers expressed a greater incidence of DR, while light and never smokers' behavior is more similar to HPV‐negative OPC.

This is the first time that a higher rate of distant recurrence has been shown in HPV‐positive OPC and that a specific analysis according to the smoking status has been performed.

## Funding

The authors have nothing to report.

## Ethics Statement

Each center has received ethical approval from its own committee and all the patients included have signed the informed consent.

## Conflicts of Interest

The authors declare no conflicts of interest.

## Data Availability

The data that support the findings of this study are available from the corresponding author upon reasonable request.
